# How Zambia reduced inequalities in under-five mortality rates over the last two decades: a mixed-methods study

**DOI:** 10.1186/s12913-023-09086-3

**Published:** 2023-02-20

**Authors:** Choolwe Jacobs, Mwiche Musukuma, Brivine Sikapande, Ovost Chooye, Fernando C. Wehrmeister, Ties Boerma, Charles Michelo, Andrea K. Blanchard

**Affiliations:** 1 School of Public Health, University of Zambia, Box 50110, Lusaka, Zambia; 2 Ministry of Health, Lusaka, Zambia; 3 Federal University of Pelotas, Pelotas, Brazil; 4 University of Manitoba, Winnipeg, Canada

**Keywords:** Under-five mortality, Inequalities, Health equity, Child health, Mixed-methods, Policy analysis, Health systems

## Abstract

**Background:**

Zambia experienced a major decline in under-five mortality rates (U5MR), with one of the fastest declines in socio-economic disparities in sub-Saharan Africa in the last two decades. We aimed to understand the extent to which, and how, Zambia has reduced socio-economic inequalities in U5MR since 2000.

**Methods:**

Using nationally-representative data from Zambia Demographic Health Surveys (2001/2, 2007, 2013/14 and 2018), we examined trends and levels of inequalities in under-five mortality, intervention coverage, household water and sanitation, and fertility. This analysis was integrated with an in-depth review of key policy and program documents relevant to improving child survival in Zambia between 1990 and 2020.

**Results:**

The under-five mortality rate (U5MR) declined from 168 to 64 deaths per 1000 live births between 2001/2 and 2018 ZDHS rounds, particularly in the post-neonatal period. There were major reductions in U5MR inequalities between wealth, education and urban–rural residence groups. Yet reduced gaps between wealth groups in estimated absolute income or education levels did not simultaneously occur. Inequalities reduced markedly for coverage of reproductive, maternal, newborn and child health (RMNCH), malaria and human immunodeficiency virus interventions, but less so for water or sanitation and fertility levels.

Several policy and health systems drivers were identified for reducing RMNCH inequalities: policy commitment to equity in RMNCH; financing with a focus on disadvantaged groups; multisectoral partnerships and horizontal programming; expansion of infrastructure and human resources for health; and involvement of community stakeholders and service providers.

**Conclusion:**

Zambia’s major progress in reducing inequalities in child survival between the poorest and richest people appeared to be notably driven by government policies and programs that centrally valued equity, despite ongoing gaps in absolute income and education levels. Future work should focus on sustaining these gains, while targeting families that have been left behind to achieve the sustainable development goal targets.

**Supplementary Information:**

The online version contains supplementary material available at 10.1186/s12913-023-09086-3.

## Background

Globally, mortality among children under five years of age declined considerably over the decades. However, disparities remain between regions, with low- and middle-income countries being more disadvantaged compared to high-income countries [1]. For instance, in 2018, the under-five mortality rate (U5MR) in low- and middle-income countries was estimated at 59 deaths per 1000 live births, compared to 5 deaths per 1000 live births in high-income countries [2]. In sub-Saharan Africa, U5MR remained particularly high compared to other regions at 76 per 1000 live births in 2019, which is still far from the Sustainable Development Goal (SDG) target of less than 25 deaths per 1000 live births by the end of 2030 [2].

Despite having comparatively higher U5MR, sub-Saharan Africa has achieved notable reductions over the past two decades [3]. Zambia experienced nearly a three-fold reduction of U5MR between 2000 and 2020 (from 156 to 61 deaths per 1,000 live births) [4], accompanied by declining socio-economic and regional disparities [5, 6]. Specifically, Zambia has been among the fastest to reduce wealth-related inequalities in U5MR in sub-Saharan Africa according to comparable demographic health surveys (DHS) over the last 20 years (see Supplementary Figs. 1 and 2) [7, 8].

Global estimates of the causes of under-five deaths in Zambia show that mortality due to acute respiratory disease (such as pneumonia) made the largest contribution to the all-cause mortality decline (21.2% of the total decline) between 2000 and 2020 [9]. Notable contributions were also made by reducing deaths due to malaria (19.6%), human immunodeficiency virus/acquired immune deficiency syndrome (HIV/AIDS) (18.0%), and diarrheal diseases (16.0%). Reducing deaths due to measles, neonatal causes (sepsis, prematurity, birth trauma) and meningitis contributed 7.4%, 7.2% and 4.1% respectively to the overall U5MR declines [9]. Other published studies on causes of death among children under five years old in Zambia are based on verbal autopsies at a single time point, usually based in a facility except the 2010 and 2015/16 SAVVY surveys, but found similar main causes for under-five deaths including pneumonia, diarrhea and other infections (with sepsis or other infections high also among newborns, as much as birth asphyxia or other intrapartum causes), as well as malaria and HIV/AIDS among others [10–13].

Several studies have provided insights into a range of factors contributing to the overall progress in child survival in Zambia, or examined the policy reforms towards specific health services and Universal Health Coverage (UHC), many with a focus on financial protection [5, 14–17]. However, more evidence on the range of health policy and systems changes that Zambia made to address inequalities in child survival over the last two decades is needed [16, 17]. There are few studies bringing together quantitative trends on child health outcomes and related intervention coverage and socio-economic conditions, with health policy and systems analysis that focuses on equity [14]. We examined Zambia’s progress in child survival through an equity lens, focusing on the contribution of health policy and systems reforms to implement high impact programmes and particularly to reach disadvantaged populations, while considering how socioeconomic development may have also affected inequalities.

## Methods

Zambia had a population of about 18 million in 2020. It reached lower-middle income country status in 2011 (which was however reversed to lower income in 2022). Income inequality in Zambia is one of the highest in sub-Saharan Africa (and the world) with a Gini index of 57 in 2015, with no improvement over the past two decades [18]. This mixed-methods study integrated quantitative inequality trend analysis with policy and health systems analysis.

### Quantitative data and analysis

We used population-representative data from the last four Zambia Demographic and Health Surveys (ZDHS) (2001, 2007, 2013/2014, 2018) to analyze trends in inequalities for mortality, health intervention coverage and socio-economic conditions. The data collection methods for the ZDHS are described elsewhere [19].

Under-five mortality rates were calculated using the syncmrates program in Stata 15. We obtained estimates of the number of deaths among children aged 0–59 months out of 1000 live births, among all live births in the ten years preceding each round of the ZDHS. We also stratified the U5MR in each ZDHS round (2001–2018) by household wealth quintile using cross-tabulations. The wealth index was adopted to examine inequalities, based on DHS’ previously-computed principal component analysis of dwelling materials, access to utilities and household assets. The wealth index is created based on the assets for rural and urban places of residence separately, and divided into quintiles; the first quintile being classified as those within the lowest 20% of wealth index scores and the fifth quintile being those within the highest 20% of wealth index scores [20].

To assess the role of compositional changes in the socio-economic position of women in the poorest and richest wealth quintiles over time, we estimated absolute income levels by quintile for each survey. The calculation of absolute income for each percentile of distribution follows the Fink et al. (2017) definition and includes the Gini index, gross domestic product (in 2011 US dollars, power purchasing parity) and the household expenditure [21]. We then attributed a value in US dollars for the mean income of each wealth quintile (levels over time shown in Supplementary Fig. 3). Absolute education levels were also examined using the proportion of women with at least secondary education within each wealth quintile as another way to assess changes in socio-economic status among the least to most disadvantaged. The direct influence of income or education levels on health is complex, non-linear and multifactorial, and it was not within our aim or scope to uncover their direct causal influence in relation to health intervention coverage or child mortality [22, 23]. Rather, this approach to characterizing wealth groups with absolute socio-economic measures has been proposed previously as valuable to help understand whether there were improvements in a country’s socio-economic growth itself, or if not, whether improvements in health among poorer groups were rather due to intentional policies or programs that overcame the disadvantages of their lower socio-economic status or income [24, 25].

We examined inequality trends in RMNCH, malaria and HIV/AIDS intervention coverage, as well as changes in living conditions such as water and sanitation, and fertility rates between ZDHS 2001 (2007 for HIV/AIDS indicator, the first with disaggregated data) and 2018 by wealth quintile, given their known association with the main U5MR causes that reduced in Zambia in that period [26, 27].

We modified the well-established composite coverage index (CCI) to include malaria prevention as the fifth intervention area [27]. The CCI includes interventions across the continuum of care, where each stage is given equal weight as follows:

$$CCI(+malaria)= \frac{1}{5} \left(DFPSmo+ \frac{ANC4+SBA}{2}+ \frac{BCG+2\times DPT3+MSL}{4}+CAREANYD+\frac{ITNch+ITNwm+IPT2}{3}\right)$$

where:

1. Reproductive care: Demand for family planning satisfied with modern methods among currently married women in need of contraception (DFPSmo)
2. Maternal care: At least four antenatal care visits during last pregnancy (ANC4); Skilled birth attendance (SBA), among births in the last three years
3. Childhood immunization received by children aged 12-23 months: BCG vaccination (BCG); DPT vaccination 3 doses (DPT3); Measles vaccination (MSL)
4. Management of childhood illness: Care-seeking for disease among children under 5 years with symptoms of fever, diarrhea or suspected pneumonia in the last 2 weeks (CAREANYD)
5. Malaria prevention: Use of insecticide treated net for child (ITNch) and woman (ITNwm), and receipt of intermittent preventive treatment in pregnancy two doses (IPT2)

To quantify and compare trends in inequalities over time, we calculated concentration indices (CIX) and slope indices of inequality (SII). CIX is calculated as twice the area between the curve and the line of equality, based on the plot of the cumulative percentage of the sample ranked by the socio-economic variable starting with worst off on x-axis and the cumulative percentage of the health variable on the y-axis. SII is the absolute difference between the predicted outcome value of the individuals with highest and lowest wealth scores, after regressing the mid-point of the cumulative proportion of the sample in each category (using a score from 0 to 1 from most to least disadvantaged) against the outcome estimate for each category [27–29].

### Policy and health systems analysis

The policy and health systems analysis involved in-depth document review of health policy reports, guidelines and strategy documents published and implemented between 1990 to date, obtained from the Zambian Ministry of Health, World Health Organization and United Nations agencies databases. We drew on quantitative health systems data from the WHO Global Health Expenditure Database [30], analysis of the Creditor Reporting System data with the Muskoka2 method [31], the WHO Global Health Database, Ministry of Health data and Zambia’s National Health Facility Census conducted in 2005 and 2017.

To assess policies and strategies that may have contributed to reductions in under-five mortality since 2000, we adapted the Countdown to 2015 Policy and Programme Timeline Tool [32]. The Policy and Programme Timeline Tool is useful for identifying health policies, programmes and health systems changes that have been implemented in a country to improve RMNCH indicators and survival over time from 1990 to present. The tool extends across six levels including: national context, macro health systems and governance, health system building blocks, high impact policies specific to RMNCH, high impact research specific to RMNCH, and a cross-cutting component focused on partnerships and convening mechanisms [32, 33]. For this analysis, we focused on three levels that were most relevant to U5MR reduction and with available data or documents to track over time: macro-level governance and health systems environment, specific health system building blocks, and high impact policies specific to RMNCH. For each, we focused on where ‘equity’ was explicitly or implicitly incorporated as a guiding principle or ‘value’ [34, 35].

## Results

### U5MR inequalities by wealth quintile

Overall, the U5MR in Zambia declined from 168 to 64 deaths per 1000 live births between ZDHS 2001/2 and 2018 (in the ten years preceding the survey). Mortality reduced most for children aged 1–59 months (from 139 to 33 per 1000 live births respectively). To a lesser extent, neonatal deaths in the first 28 days of life reduced (from 37 to 27 per 1000 live births respectively). Perinatal mortality rate estimates hardly reduced (38 to 33 per 1000 live births), suggesting that neonatal deaths during the intrapartum period have not gone down noticeably.

**Fig. 1:**
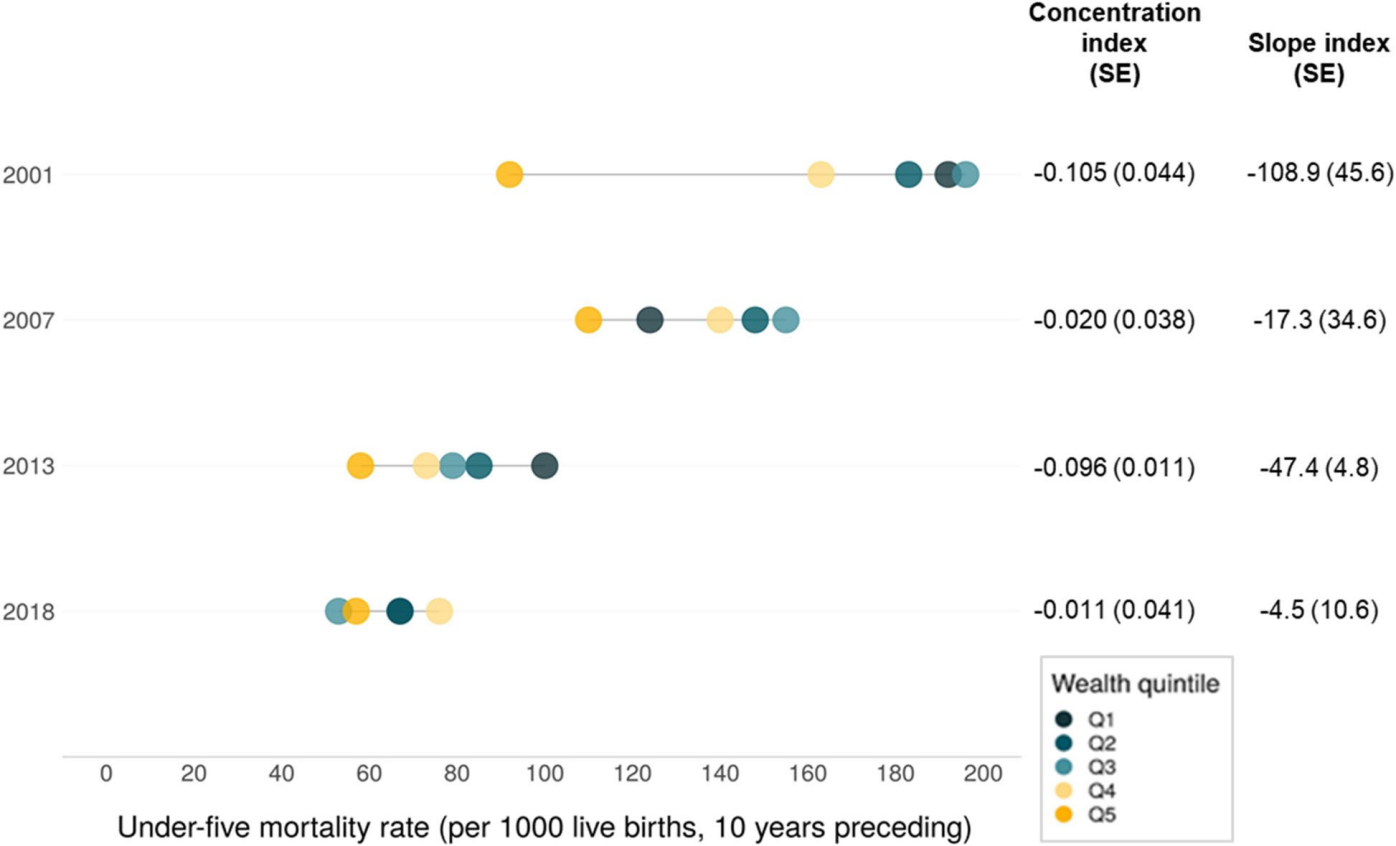
Trends in U5MR by wealth quintile, with concentration indices (CIX), slope indices of inequality (SII) and related standard errors (SE), ZDHS 2001/2, 2007, 2013/14 and 2018

Declines in U5MR were shown in all wealth quintiles in the last two decades, with a consistent pattern except for the two wealthiest quintiles in the first two surveys (Fig. 1; estimates with confidence intervals found in Supplementary Table 1). The reductions were fastest among the poorer groups in the bottom three quintiles, compared to the richest in wealth quintile 5 (92 to 57 per 1000 live births). This led to reduced inequalities in U5MR between ZDHS 2001 and 2018, reflected in a U5MR CIX that reduced from -0.11 to -0.01, and SII in U5MR from -108.9 to -4.5 deaths per 1000 live births respectively. The greatest progress was made between ZDHS 2001 and 2007, which roughly refers to the period from 2008 onward.

### U5MR inequalities and absolute socio-economic changes

**Fig. 2:**
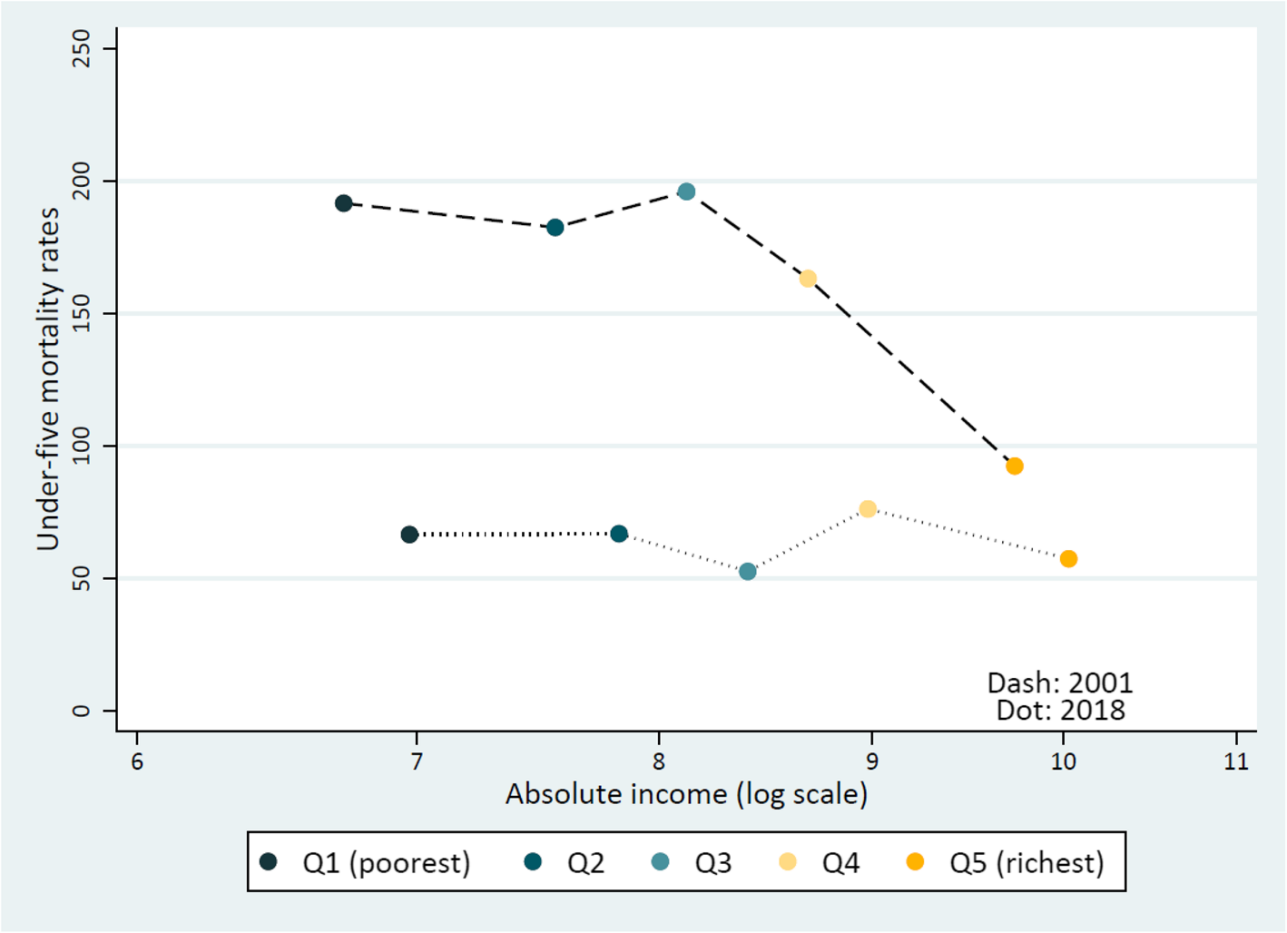
U5MR per 1,000 live births by absolute income level (log scale) in each wealth quintile, ZDHS 2001 and 2018

To understand whether inequality reductions in U5MR between wealth groups could be related to absolute changes in economic inequalities in Zambia, we attributed an absolute dollar value to each wealth quintile and plotted this against the respective under five mortality rates in the 2001 and 2018 surveys (Fig. 2). Although the mortality gap between the extremes of wealth quintiles was virtually closed by 2018, there were no major simultaneous increases in income levels among the poorest groups to explain this.

We also examined whether compositional changes in the number of women with secondary or more education (absolute education levels) among lower to higher wealth quintiles was evident alongside reductions in U5MR among these groups in the 2001 and 2018 surveys (Supplementary Fig. 4). The difference in the proportion of women with secondary or higher education between the poorest and the richest remained wide, and so did not occur at the same time as the relative decline in U5MR inequalities.

### Equity trends in health intervention coverage and socio-demographic conditions

**Fig. 3:**
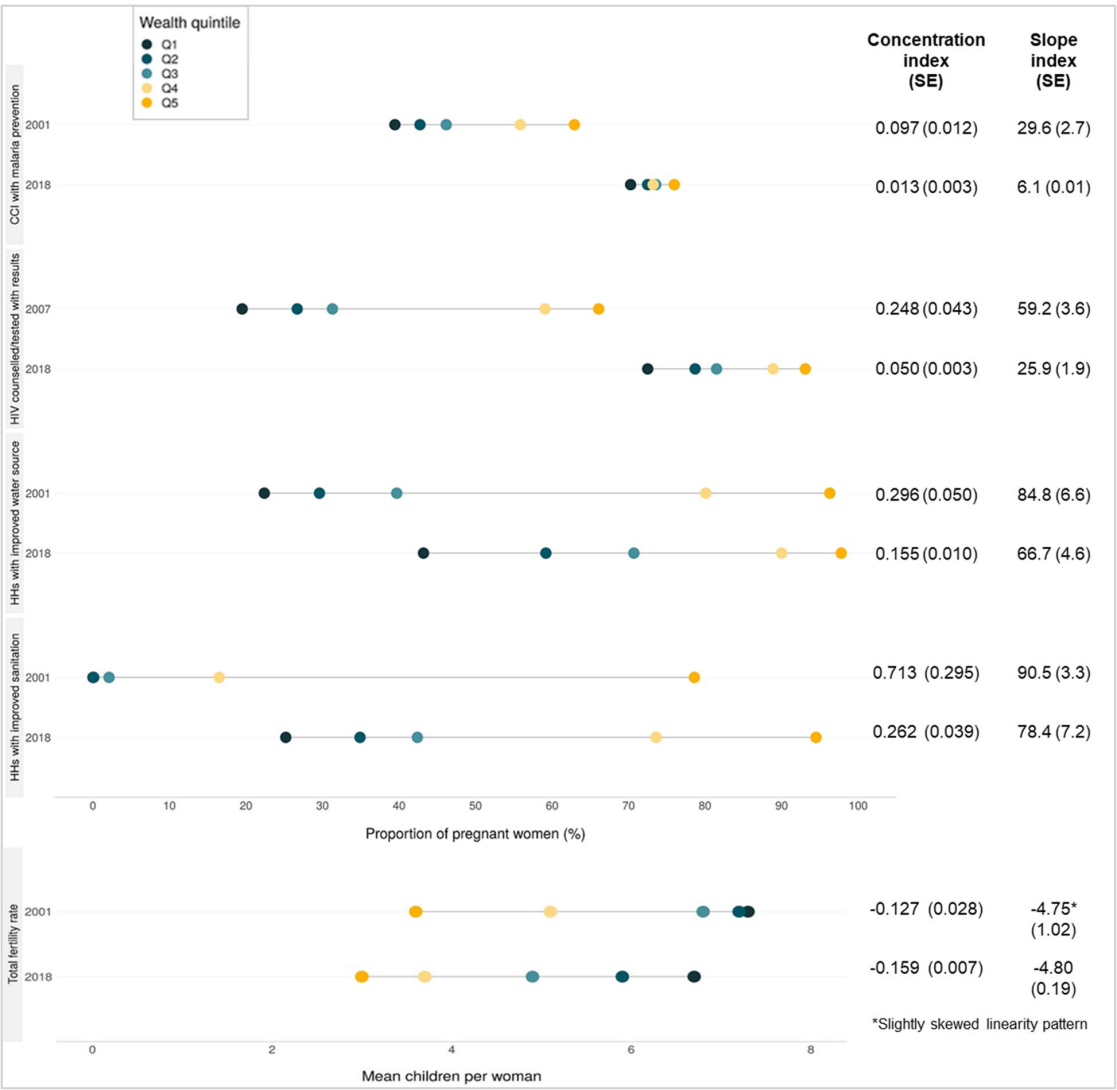
RMNCH intervention coverage, HIV/AIDS prevention, household (HH) water and sanitation, and total fertility rate by wealth quintile, with concentration indices (CIX), slope indices of inequality (SII) and related standard errors (SE), ZDHS 2001 (2007 for HIV/AIDS) and 2018

We also examined trends in wealth-related inequalities in coverage of health interventions (RMNCH and malaria control, and HIV/AIDS Prevention of Mother to Child Transmission or PMTCT), and socio-demographic conditions including water, sanitation and fertility levels, that could also be related to the major causes of U5MR that reduced. The RMNCH CCI, including malaria control, improved greatly from moderate to small wealth differences between 2001 and 2018 (CIX of 0.1 to 0.01, or SII from 30 to 6 percentage points respectively) (Fig. 3). Further, the ZDHS shows that improvements among the poorest compared to the richest groups occurred for most components in the CCI across the continuum of care; skilled birth attendance, DPT and measles immunizations, and malaria prevention interventions became particularly more equal between groups (Supplementary Fig. 5). Inequalities in HIV/AIDS prevention also improved (PMTCT, in terms of women receiving counselling, testing and related results during ANC) from wide disparities and the richer 40% at much higher levels in 2007 (no disaggregated data in 2001/2 ZDHS), to moderate disparities with much higher coverage levels for all groups in 2018 (0.25 to 0.05 for CIX; 60 to 26 percentage points in SII respectively).

The major inequalities in household access to improved water and sanitation within Zambia did not improve so noticeably. There were absolute improvements in water and sanitation access among the poorest groups, albeit from nearly no access in 2001. Meanwhile, total fertility rates reduced slightly and evenly in both poorer and wealthier groups, such that inequalities remained equivalent over time in absolute terms (SII showed a consistent gap of 4.8 fewer children among richer than poorer women) and relative terms (CIX at and then above -0.13).

### Policy and health system changes

Through our in-depth policy and health systems analysis, we identified five themes on potential key drivers that contributed to the reduced inequalities in child survival. These are summarized with the main points and counterpoints of evidence in Table 1, and subsequently explored in more detail.

**Table 1:** Summary of evidence on the contribution of key drivers to the reduction in inequalities in child survival, Zambia, 1990s-2020

**Key drivers**	**Points of evidence on contribution**	**Counterpoints**
*Policy commitment to equity in RMNCH*	• Equity targets integral in policies since 2000 • Equity strategies and targets integral element of RMNCH strategies • Comprehensive set of child health plans since 2011	• Few explicit equity indicators with targets in overall health plans and RMNCH strategies and plans
*Health financing with focus on disadvantaged groups and regions*	• Increasing reliance on public funding sources, including external funding • Increasing per capita government expenditure on RMNCH from 2002 • User removal fee policy in 2006, and less out of pocket expenses including for the poorest • Needs-based financing for districts; district performance monitoring and contracting since mid 1990s	• Dependence on external funding remains large for health sector in general and for RMNCH (%) • District performance monitoring not universal
*Multisectoral partnerships and horizontal programming*	• Sector-wide approaches (SWAp) predominant since mid-nineties • Good government coordination of external partners, working across sectors • Integration of mother and child health with community development administratively	• Less coordination and partnership across sectors in practice than policy (unless proven otherwise) • Less evidence or data to relate these directly to equitable improvements in outcomes
*Expansion of infrastructure and human resources for health (HRH)*	• Massive increase in primary health posts 2005–2017 (more than sevenfold), particularly in rural areas • Increase in density of nurses/midwives (by 40%) and physicians (by 80%)	• Health workforce density too low, while workloads have increased • Limited increase in density of secondary health centers and hospitals, and in specialists • Retention of health workers harder in the more rural and remote areas • Equity in quality of care not studied
*Involvement of community stakeholders and service providers*	• Community based outreach and mobilization strong • Multiple community organizations and cadres to engage rural and remote populations	• Mixed performance of community cadres in evidence so far

#### Policy commitment to equity in RMNCH

**Fig. 4:**
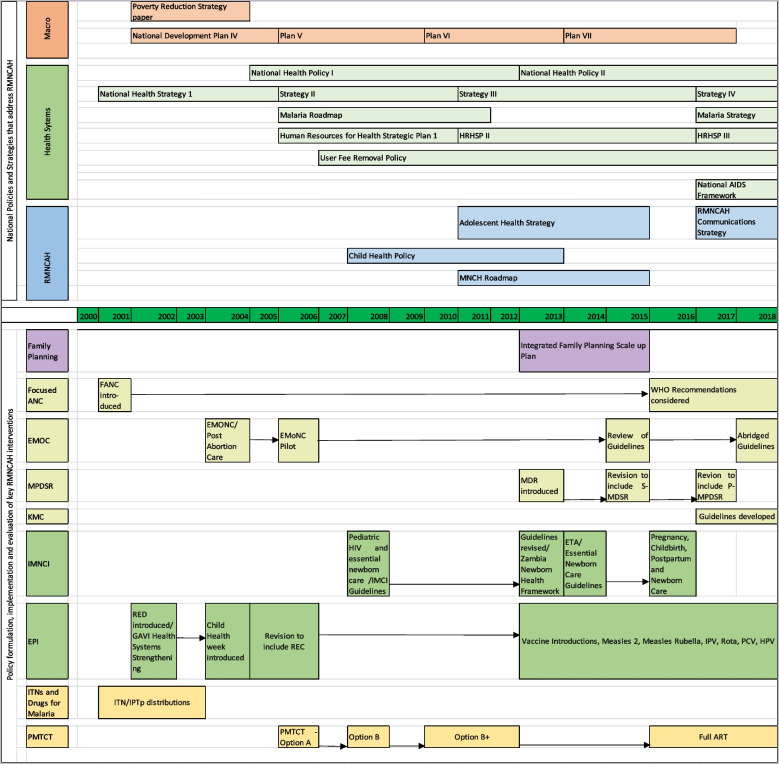
Policy and health system reforms for RMNCH and related programmes since 2000 in Zambia

Zambia’s health sector policy reforms since the 1990s have been guided by the consistent vision to, “provide equity of access to cost-effective, quality health care as close to the family as possible” [36–38]. These health sector reforms have increasingly taken a holistic approach to health sector development that consistently included a cross-cutting goal of reducing inequities between socio-economic groups and regions. As shown in Fig. 4, at the macro-level is the National Development Plan, the country’s ‘blue print’ for eliminating poverty and accelerating development efforts towards the vision 2030 of ‘leaving no one behind’, which has been developed and revised since 2002. Health sector policies since 2000 focused on successively improving health systems generally, and RMNCH programmes specifically, including formulation, implementation, and evaluation of specific interventions across the continuum of care. Since the early 2000s, the health sector embedded these plans aimed at strengthening and prioritizing RMNCH services into its broader National Development Plans, Sector Strategic Plans and Programme Strategic plans.

In line with these reforms, health systems improvements occurred over time in numerous ways that may have contributed to equity in child health outcomes by improving access to RMNCH, HIV/AIDS and malaria prevention services for rural, poorer and less educated groups. Policies and guidelines such as the National Health Strategy (2001), the Human Resources for Health Strategic Plans and user fee removal policy (both 2006) were developed, reviewed and updated over time to strengthen the health system, and specifically to improve the manner in which RMNCH services were delivered.

Notably, since 2000, child health received more programmatic emphasis through the addition of Integrated Management of Childhood Illnesses (cIMCI), integrated Community Case Management (iCCM), and the Expanded Program on Immunization (EPI). The Child Health Policy and MNCH Roadmap were developed in 2008 and 2011 respectively. Since 2006, Prevention of Mother to Child Transmission (PMTCT) expanded from Option A, B, B + towards full antiretroviral therapy in 2016. Since 2001, malaria control has been strengthened significantly (Fig. 4).

#### Needs-based health financing with focus on disadvantaged groups and regions

**Fig. 5:**
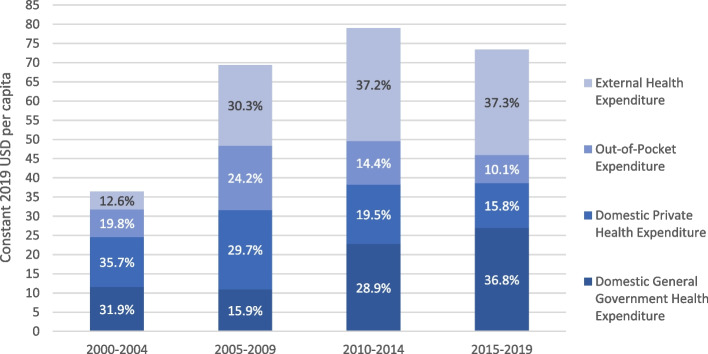
Five-year general health expenditure (USD per capita, and proportion of total) in Zambia by source between 2000–19, Global Health Expenditure Database (GHEx)

Financing increased for health from government and donors, more than private and out-of-pocket expenses, thus likely having a pro-equity impact. Overall government health expenditure continually increased both in absolute terms (from 12 US Dollars, 2019 constant) per capita between 2000–2004 to 27 United States Dollars (USD) per capita between 2015–19 (Fig. 5). This constituted slightly more of the rising total over time (32% up to 37% respectively). External (donor) expenditure also increased from 5 to 27 USD per capita (2019 constant) between these time periods (and from 13 to 37% respectively), but remained similar in 2015–19 as in 2010–14. Comparatively, out of pocket expenditure remained at 7 USD per capita, which reduced relative to other sources from around 20% to 10% of the total. This may reflect that the user fee removal policies have been contributing to fewer poorer families paying out of pocket for health services including for RMNCH [39, 40]. Private health expenditure (e.g. insurance) also declined from around 36% to 16% of the total.

For RMNCH specifically, data available from national sub-accounts (analysed with the Muskoka2 method) showed that government expenditure per capita increased (and remained consistent from donors), particularly for HIV/AIDS and malaria, but also maternal health, family planning and immunization [31]. External funding for RMNCH in Zambia continuously increased between 2002 and 2017, from 51 to 233 million USD (constant 2016).

Decentralized planning and needs-based financing appeared to be important in strengthening equity-oriented primary health care in Zambia according to some past evidence [14, 41, 42]. Annual action plan budgets at facility and district levels with the involvement of District Health Management Teams facilitated needs-based disbursement of funds [14, 43]. To provide direct financing to districts and encourage bottom-up planning and management, a needs-based financing formula and national system-wide performance-based contracting (PBC) through the Central Board of Health were put in place in the mid-1990s [14, 41]. The Central Board of Health was an autonomous body responsible for service provision contracted by the Ministry of Health as the purchaser of services, but was dissolved in 2006 with the Ministry of Health taking responsibility for provision again. The decentralized approach has continued in the form of district-level performance monitoring, with performance-based contracting between 1996 and 2006, which was found to have improved equity in access to MNCH services through collective planning based on high-level support [14]. This was followed by results-based financing (RBF) in several districts since 2008, which was found to have success, and was scaled up more widely in 2012 to improve RMNCH outcomes [14, 16, 44].

#### Multi-sectoral partnerships and horizontal programming

Strong governance with continued commitment to equitable improvements in health were pursued through a Sector-Wide Approach (SWAp) since the 1990s, which brought together the government and other stakeholders in ensuring efficient and effective utilization of programme resources [14, 45]. Earlier reviews have shown that this did not achieve optimal efficiency and harmonization, but indicate there was high-level support to pursue this [45]. Policies and programs also reflected a renewed focus on primary health care particularly since 2008 in line with the Declaration of Ouagadougou on primary health care and health systems. Further, Zambia’s health system reforms have emphasized multi-sectoral collaboration and horizontal programming. This is guided by international recommendations and resolutions including the children’s rights to health, the road map for Millennium Development Goal 4, UHC, primary health care and evidence-based RMNCH interventions [41, 43, 46]. In 2012, the Ministry of Health was split whereby the mother and child health function was taken to the Ministry of Community Development and Maternal and Child Health, with the Ministry of Health focusing more on health policy, research and curative care [41, 43]. This shift lasted until 2015 when the primary health care functions were transferred back to the Ministry of Health. However, despite transfer of functions, there has been continued collaboration of the two ministries through the implementation of the National Development Plans. The Zambian government has also worked in partnership with external donors to align priorities and coordinate resources, planning, and service delivery across sectors to implement health programmes for RMNCH along with malaria prevention and HIV/AIDS, community development, literacy, nutrition, and water, sanitation and hygiene [46].

#### Expansion of infrastructure and human resources for health

The government has also aimed to improve infrastructure and human resources for health. Policies mandated the construction of health posts, mini-hospitals and first level hospitals especially in rural areas. The country aimed to ensure that over 80% of households in the urban and rural areas are within 5 km radius of a health facility. The National Health Facility Census in 2017 showed that the density of health posts, which primarily serve rural populations, increased substantially from 8 to 61 per 10,000 population between 2005 and 2017. However, the density of larger public facilities, such as health centers and hospitals, did not increase over time.

**Fig. 6:**
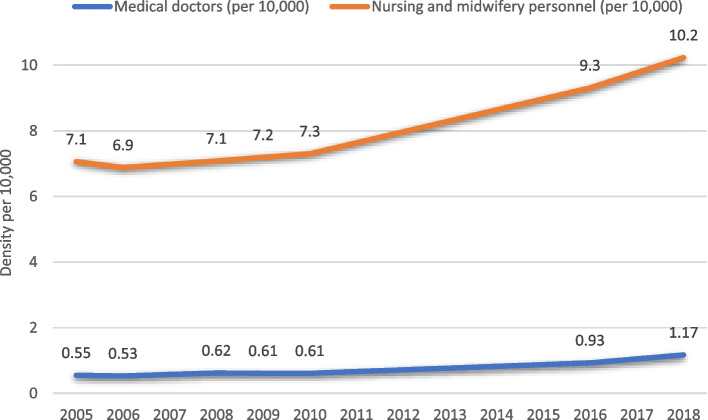
Changes in nurses/midwives (WHO Global Health Database and Ministry of Health data) per 10,000 population from 2005 to 2018

Through the Human Resources for Health Strategic Plans (the first for 2011–2017), Zambia aimed to improve production of core health workers from training institutions, as health worker densities were one of the lowest in the region, especially in rural settings [47]. Between 2005 and 2018, the estimated density of nurses and midwives increased from 7.1 to 10.2 per 10,000 population (Fig. 6), and was 11.3 in 2019 according to Ministry of Health data. The density of physicians increased from 0.5 to 0.9 per 10,000 from 2006 to 2016 according to WHO data, and to 1.2 in 2019 in Ministry data [48, 49]. The strategic plans specifically focused on recruitment and distribution of critical cadres in disadvantaged areas, also engaging private partnerships, and task shifting to community-based health workers to enhance outreach and linkages to rural facilities. Efforts to increase retention of health workers in rural areas had more limited results, requiring further attention [50, 51]. Some evidence also suggests that expanding mandates particularly for HIV/AIDS and PMTCT have also increased workloads of health staff, while numbers of health workers have not risen in parallel [52].

#### Involvement of community stakeholders and service providers

Finally, Zambia has pursued efforts to improve equity in RMNCH and related programmes by enhancing community stakeholder ownership and involvement in service delivery at village level. This has included implementing Child Health Weeks to provide community-based outreach for immunizations and ITN distribution to prevent malaria, among others [43]. Planning and provision of RMNCH services for rural and remote populations also involves Neighborhood Health Committees, Safe Motherhood Action Groups (SMAGs), Community Health Workers (CHWs), and Community Health Assistants (CHAs). A Community Health Worker strategy was put in place since 1983 to address a shortage in human resources for health by training a community cadre living in and selected by the community to provide primary health care. More recently in 2012, the CHA programme was established, and the government now provides this new cadre with one year of formalized training and a monthly salary. The CHAs have varied responsibilities in delivering RMNCH interventions, including to counsel and monitor pregnant women, link them to services, and provide cIMCI and iCCM (which had been introduced in 2008 and scaled up since 2013). CHWs, SMAGs and CHAs provide community-based counselling and outreach for family planning, birth planning and linkages to ANC where PMTCT is provided, counselling on early and exclusive breastfeeding, malaria prevention and management, and diarrhea treatment and care-seeking [43, 53–55].

## Discussion

Zambia has made remarkable progress in improving child survival, evidenced by the substantive decline in the U5MR. Our equity trend analyses showed that the country has achieved great reductions in U5MR inequalities, through faster improvements among the poorest socio-economic groups, at a pace that is among the fastest in the region. There were no simultaneous improvements in absolute income or education among the poorer relative to richer groups in Zambia in this period, nor in wealth inequalities for access to water and sanitation, and fertility levels. Conversely, inequalities in intervention coverage for RMNCH, malaria and HIV/AIDS prevention closed noticeably between wealth groups as levels among poorer groups increased. Our policy and systems analyses uncovered an array of interrelated health sector reforms that could have contributed to the equitable improvements that Zambia achieved in child survival.

Our finding that U5MR reductions among the poorest groups occurred without substantial concomitant improvements in their socio-economic positions over time (in terms of absolute income and secondary education levels) adds to previous research showing reduced U5MR inequalities in Zambia [56–58]. Some other studies argue that the influence of mother’s wealth or education on U5MR can be indirect, by contributing to other factors such as higher women’s empowerment, improving health status and reproductive decision-making, and health service utilization by improving knowledge and awareness of the services [56, 58–60]. However, a couple of other studies found a strong independent protective effect of maternal education and wealth on U5MR in other countries [1, 61]. One study found that U5MR reduction in Zambia was most strongly associated with coverage of a composite of health interventions that had been rapidly scaled-up (malaria prevention, PMTCT, exclusive breastfeeding and pentavalent immunization, as well as DPT3), even after adjusting for socio-economic status and underweight among children (which were not significant), while another multivariate analysis showed that the mother’s wealth or education in 2018 were no longer important factors for U5MR [62, 63].

There has been a declining number of HIV/AIDS and malaria infections among children since 2000 in Zambia, consistent with a decreased proportion of child deaths due to these causes [64]. This is likely related to the relatively high investment for addressing HIV/AIDS and malaria, and integration with RMNCH services, resulting in an expanded number of children with HIV/AIDS who are receiving antiretroviral therapy [4, 64–66]. The expanded responsibilities of the CHWs as a critical first line of defense against malaria for diagnosing and treating malaria cases in their communities, delivering mass drug administration of malaria prophylaxis, and collecting data has also likely contributed to reduction of malaria-related deaths in Zambia [67].

The finding that the large wealth-based inequality reduction in U5MR was not related to absolute changes in income or education levels between groups is understandable given uneven economic change in this period. The Zambia SDG national review reported that multi-dimensional poverty has reduced moderately from 69 to 59 between 2016 and 2020 [64]. Economic growth was initially fast, with a growth rate from 7% between 2005 and 2014, slowing to 4% in 2018, while socio-economic inequalities remained very large [18]. This points to the particular importance of the intentional changes Zambia made to expand access to RMNCH services, malaria and HIV/AIDS prevention among poorer families for equitably improving child survival.

The policies and guidelines that Zambia developed, reviewed and updated such as the national health strategy, human resources for health strategy, removal of user fees policy and child health policy had clear aims for strengthening the health system specifically to improve the manner in which RMNCH services were delivered to ensure equitable access. We conclude that the policy reforms and resulting health systems strengthening such as removal of user fees to reduce the cost barrier for accessing services, combined with increasing physical access by moving these services closer to families particularly at the primary care level to provide immunization and outreach for ANC with PMTCT and malaria prevention, were significant contributors to reducing inequalities in RMNCH intervention coverage and child survival. Available data in our study shows that out-of-pocket expenditure reduced since 2000, indicating that user fee removal in 2006 could have helped the poorest groups in better accessing RMNCH services. Studies evaluating user fee removal together show that this policy promoted a shift from care-seeking at private to more public health care, and that a very small proportion of people have incurred user fees since this policy was enacted [16, 39, 40, 68–70]. Among them, catastrophic health expenditure was still higher among those in rural and remote or poorer populations, possibly because of higher informal payments such as for drugs at private pharmacies/clinics or transport, due to differential quality of and distance to facilities of Zambia [14, 16, 39, 70, 71]. Efforts continue towards removing financial barriers through the enactment of a national health insurance scheme in 2019, whose contributions to universal health coverage would be worth monitoring [72]. To improve availability of health facilities, researchers found that while gaps remain in rural and remote areas, there had been increases in rural health posts closer to families similar to this study’s findings [5, 73]. Though little evidence exists on equity in quality of services, one study found that urban health facilities generally had higher competence for delivery care, yet another showed how care in primary facilities had higher acceptability among rural groups than higher level facilities [73, 74].

Further, some evidence shows that community-based interventions through the CHAs and SMAGs have contributed to improving coverage of malaria prevention, immunization, counselling on antenatal, delivery and postnatal care interventions particularly for remote and poorer families [43, 53–55]. This was attributed in part to their roles in improving the acceptability and related utilization of services for RMNCH and HIV prevention interventions, and care seeking for childhood illness within the community or at primary health centres [43, 53, 54]. Still, some literature showed gaps in retention, recruitment, training and supervision for CHWs or CHAs, especially early in the pilot stages [75–77]. Others indicate that community programs could not overcome barriers like long distance to or poor quality of services to which they referred families, particularly for delivery and postnatal care, which deserves further study to better reduce inequalities in maternal and newborn health [43, 53, 54]. Recently, Zambia has also been recognized as an ‘exemplar’ in vaccine delivery based on major progress in DPT3 coverage over time, which was not attributed to economic growth but rather its intentional efforts for improving community access, facility readiness and relatedly improving demand for child vaccination [42]. They emphasized the importance of a bottom-up approach to program planning, reporting, managing and evaluating at all levels, and involving community actors not only in service delivery but also in monitoring and adapting program activities [42]. Others have noted the need for more intentional integration of community-based groups and health workers into health systems frameworks like the building blocks, which would further formalize support to the multifaceted roles they have filled to improve equity in RMNCH in Zambia [78].

There were limitations in this study. The study’s scope did not allow us to explore causation or indeed attribution through regression modelling, or triangulation with other methods like key informant interviews, nor did it allow us to look into quality of care, which would be valuable next steps. Direct comparison of U5MR and coverage equity trends was not feasible, given the need to measure U5MR 10 years preceding the survey to allow sufficient sample sizes for disaggregation, especially in the two earlier ZDHS rounds. Further, data on health financing and human resources were more available in recent years. Global Health Expenditure data reflect a combination of national health accounts data from the Ministry of Health as well as interpolation or modelled data to fill in estimates for missing time points. Future analyses of district-level variations in and relationships between mortality, RMNCH and related health system indicators would be valuable in Zambia, similar to studies in other countries [79, 80] Remaining health systems gaps were identified in other studies such as in supply chain, few specialists for emergency obstetric and newborn care, and deployment in the most remote areas, which deserve more attention through mixed-methods research including key informant interviews [5, 41, 81]. As child deaths in Zambia are increasingly concentrated in the intrapartum and early newborn period, it would be particularly important to study the barriers to improving equity in quality of obstetric and newborn care through mixed-methods.

## Conclusion

Overall, our analyses showed that Zambia achieved major improvements in reducing inequalities for under-five mortality and coverage of RMNCH as well as malaria and HIV prevention interventions, which largely stem from intentional health sector changes in a wide range of policies, strategies and investments at the community and facility levels that centrally valued equity. Still, more research is needed on how child health service access and quality has been improved equitably and what barriers remain. There is a need to sustain successful health strategies and policies through continued investment in comprehensive primary health care with an equity lens. To reach the goal of leaving no one behind in Zambia, future efforts must ensure that equity analyses on RMNCH indicators continue, to develop a strong knowledge base with the power to track progress towards universal health coverage and ultimately save lives over the medium to long term.

## Supplementary Information

**Additional file 1: Supplementary figure 1.** Absolute average annual change in under-five mortality rate among the richest by the poorest quintile (red line is average of included countries), DHS 1996-2019. **Supplementary figure 2.** Slope index of inequality (with 95% confidence intervals) in under-five mortality rate between the richest to poorest wealth quintiles (red line indicates average of included countries), DHS 1996-2019. **Supplementary figure 3.** Trends in absolute income (in 2011 US dollars, purchasing power parity) for each wealth quintile in Zambia, ZDHS 2001 to 2018. **Supplementary table 1.** Under-five mortality rate (per 1000 live births, in the 10 years preceding survey) and confidence intervals (95% CI) overall, and by wealth quintile and rural-urban residence, ZDHS 2001/2, 2007, 2013/14 and 2018. **Supplementary figure 4.** Under-five mortality rates by absolute education (secondary or more) in each wealth quintile, ZDHS 2001 and 2018. **Supplementary figure 5**. Changes in composite coverage index components for the poorest (Q1) and richest (Q5) wealth quintiles in Zambia, ZDHS 2001 and 2018.

## Data Availability

Data is available publicly online upon request: https://dhsprogram.com/data/.

## Abbreviations

ANC4: Antenatal Care—four or more visits
BCG: Bacille Calmette-Guerin
CAREANYD: Care-seeking for any childhood illness
CCI: Composite Coverage Index
CHA: Community Health Assistant
CHW: Community Health Worker
CI: Confidence Interval
cIMNCI: Comprehensive Integrated Management of Childhood Illnesses
CIX: Concentration Index
DFPSmo: Demand for family planning satisfied with modern methods
DPT3: Diphtheria-Pertussis-Tetanus three doses
EmOC: Emergency Obstetric Care
EPI: Expanded Program on Immunization
GHEx: Global Health Expenditure Database
HIV/AIDS: Human Immunodeficiency Virus/Acquired Immune Deficiency Syndrome
HRHSP: Human Resources for Health Strategic Plan
iCCM: Integrated Community Case Management
IPT2: Intermittent Preventive Treatment in pregnancy two doses
ITN: Insecticide Treated Net, for child (ch) and woman (wm)
MSL: Measles and Rubella vaccination
MPDSR: Maternal and Perinatal Death Surveillance and Response
PMTCT: Prevention of Mother-To-Child Transmission
RMNCH: Reproductive, Maternal, Newborn and Child Health
SBA: Skilled Birth Attendance
SDG: Sustainable Development Goal
SE: Standard Error
SII: Slope Index of Inequality
SMAG: Safe Motherhood Action Group
SWAp: Sector-Wide Approach
U5MR: Under-Five Mortality Rate
USD: United States Dollars
ZDHS: Zambia Demographic and Health Survey
