# Supplementary material for: How Zambia reduced inequalities in under-five mortality rates over the last two decades: a mixed-methods study
Source: BMC Health Serv Res. 2023 Feb 20;23:170. doi: 10.1186/s12913-023-09086-3 (PMC9940360; doi:10.1186/s12913-023-09086-3)
Supplement: Supplementary file 1 — Additional file 1: Supplementary figure 1. Absolute average annual change in under-five mortality rate among the richest by the poorest quintile (red line is average of included countries), DHS 1996-2019. Supplementary figure 2. Slope index of inequality (with 95% confidence intervals) in under-five mortality rate between the richest to poorest wealth quintiles (red line indicates average of included countries), DHS 1996-2019. Supplementary figure 3. Trends in absolute income (in 2011 US dollars, purchasing power parity) for each wealth quintile in Zambia, ZDHS 2001 to 2018. Supplementary table 1. Under-five mortality rate (per 1000 live births, in the 10 years preceding survey) and confidence intervals (95% CI) overall, and by wealth quintile and rural-urban residence, ZDHS 2001/2, 2007, 2013/14 and 2018. Supplementary figure 4. Under-five mortality rates by absolute education (secondary or more) in each wealth quintile, ZDHS 2001 and 2018. Supplementary figure 5. Changes in composite coverage index components for the poorest (Q1) and richest (Q5) wealth quintiles in Zambia, ZDHS 2001 and 2018. [file 12913_2023_9086_MOESM1_ESM.docx]

**Supplementary materials**


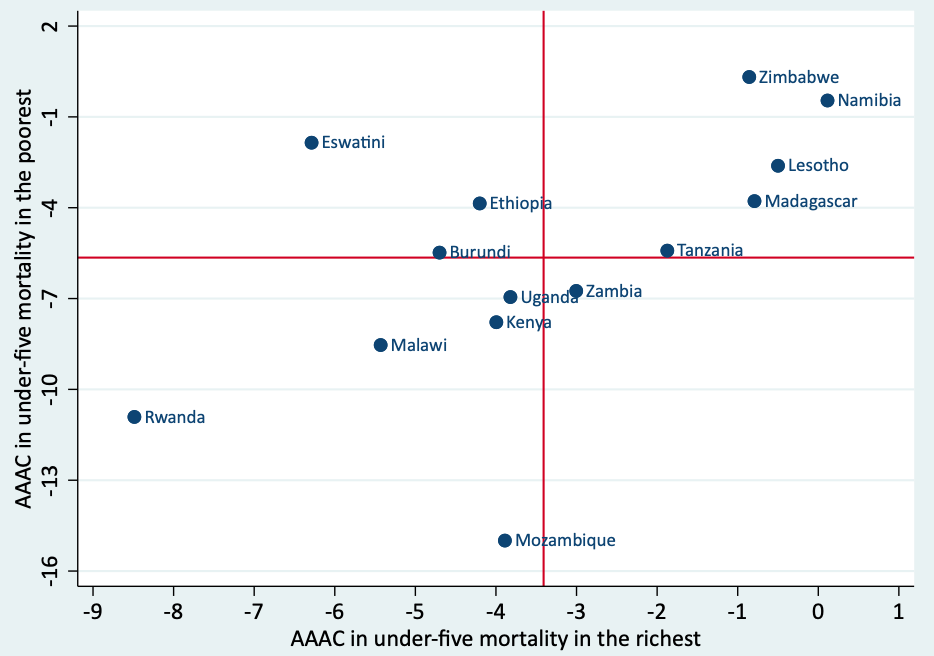


Supplementary Figure 1: Absolute average annual change in under-five mortality rate among the richest by the poorest quintile (red line is average of included countries), DHS 1996-2019


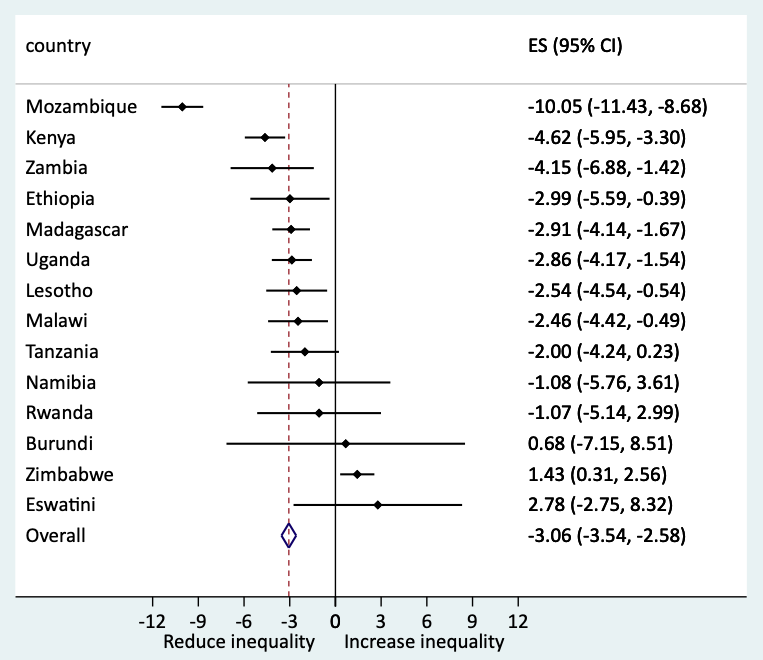


Supplementary Figure 2: Slope index of inequality (with 95% confidence intervals) in under-five mortality rate between the richest to poorest wealth quintiles (red line indicates average of included countries), DHS 1996-2019

Supplementary Figure 3: Trends in absolute income (in 2011 US dollars, purchasing power parity) for each wealth quintile in Zambia, ZDHS 2001 to 2018

Supplementary Table 1: Under-five mortality rate (per 1000 live births, in the 10 years preceding survey) and confidence intervals (95% CI) overall, and by wealth quintile and rural-urban residence, ZDHS 2001/2, 2007, 2013/14 and 2018

|  | **2001/2** | | **2007** | | **2013/14** | | **2018** | |
| --- | --- | --- | --- | --- | --- | --- | --- | --- |
|  | **U5MR** | **95% CI** | **U5MR** | **95% CI** | **U5MR** | **95% CI** | **U5MR** | **95% CI** |
| **Overall** | 168 | 161-175 | 137 | 130-144 | 81 | 77-85 | 64 | 60-69 |
| **Wealth quintile 1** | 192 | 177-206 | 124 | 110-138 | 100 | 91-108 | 67 | 59-74 |
| **Wealth quintile 2** | 183 | 167-198 | 148 | 133-162 | 85 | 77-92 | 67 | 59-75 |
| **Wealth quintile 3** | 196 | 180-211 | 155 | 140-171 | 79 | 71-87 | 53 | 45-60 |
| **Wealth quintile 4** | 163 | 147-180 | 140 | 124-156 | 73 | 63-83 | 76 | 60-93 |
| **Wealth quintile 5** | 92 | 78-107 | 110 | 92-128 | 58 | 48-68 | 57 | 45-70 |
| **Rural** | 182 | 173-191 | 139 | 131-147 | 85 | 80-90 | 62 | 57-66 |
| **Urban** | 140 | 128-152 | 132 | 119-145 | 72 | 66-79 | 68 | 58-79 |


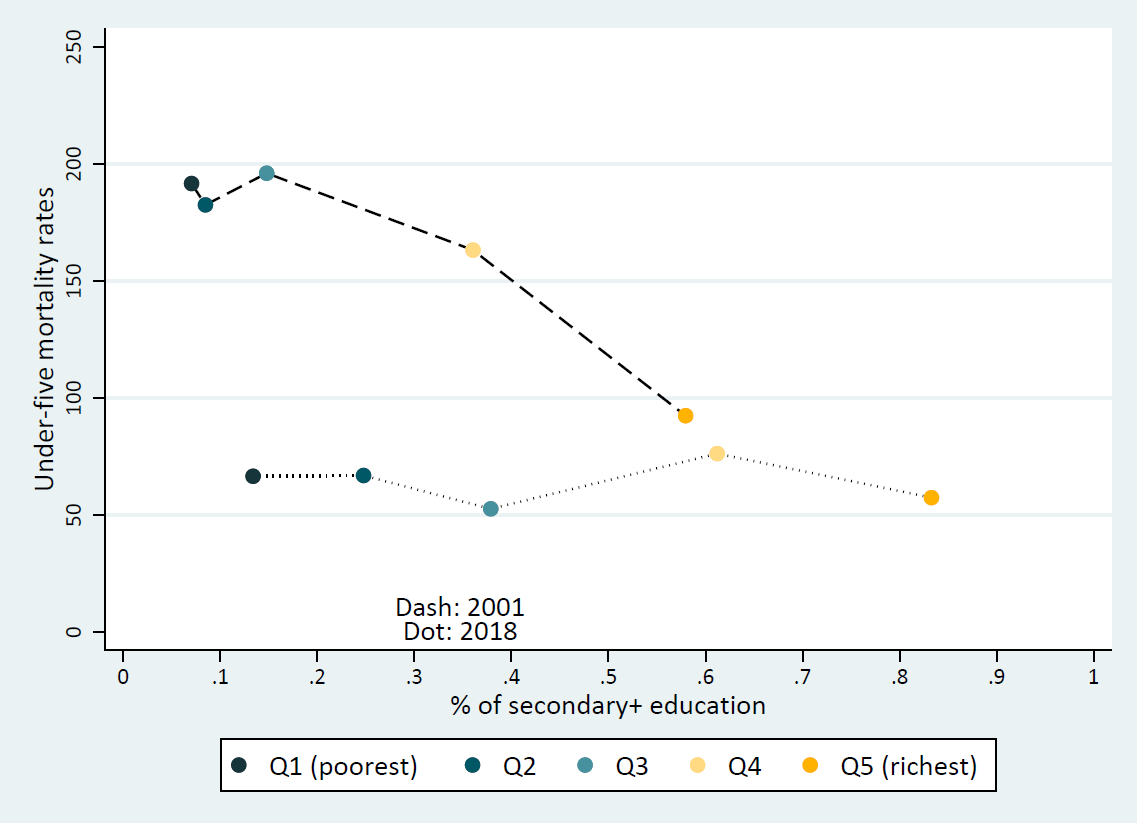


Supplementary Figure 4: Under-five mortality rates by absolute education (secondary or more) in each wealth quintile, ZDHS 2001 and 2018

[**Notes:** IPTp was not measured in ZDHS 2001/2; it was imputed at 1% for both groups as it was assumed to be low for both groups at this time. ANC 4+ was unexpectedly high in 2001, as it fell in 2007 and then sequentially improved in 2013 and 2018, possibly due to differences in how the question was framed in 2001 and later.]

Supplementary Figure 5: Changes in composite coverage index components for the poorest (Q1) and richest (Q5) wealth quintiles in Zambia, ZDHS 2001 and 2018
